# Morpho-physiological and biochemical responses and genomic stability of sweet corn (*Zea Mays* L. saccharata) to potassium ferrite nano-fertilizer

**DOI:** 10.1186/s12870-026-08131-7

**Published:** 2026-02-12

**Authors:** Dina M. Salama, M. E. Abd El-Aziz, Samira A. Osman, Mohamed S. A. Abd Elwahed, E. A. Shaaban

**Affiliations:** 1 Vegetable Research Department, National Research Centre, P.O. 12622, 33 El Bohouth St., Dokki, Giza, Egypt; 2 Polymers and Pigments Department, National Research Centre, P.O. 12622, 33 El Bohouth St., Dokki, Giza, Egypt; 3 Genetics and Cytology Department, National Research Center, P.O. 12622, 33 El Bohouth St., Dokki, Giza, Egypt; 4 Botany Department, National Research Centre, P.O. 12622, 33 El Bohouth St., Dokki, Giza, Egypt; 5 Pomology Department, National Research Centre, P.O. 12622, 33 El Bohouth St,. Dokki, Giza, Egypt

**Keywords:** *Zea Mays* L., Nano-fertilizer, Production, Biochemical contents, ISSR, Genomic stability

## Abstract

A promising method for enhancing crop performance and improving nutrient use efficiency while minimizing environmental impacts is the use of nano-fertilizers. Herein, potassium ferrite nanoparticles (KFeO_2_-NPs) as a source of potassium, synthesized via a sol-gel method, were applied as foliar sprays at varying concentrations (0, 25, 50, 75, and 100 mg/L) to white and yellow cultivars of sweet maize (*Zea mays* L. saccharata). It was sown on 1st March during the 2023 and 2024 seasons in clay soil located in Shebin El-Kom, El-Menoufia Governorate, Egypt. Over two growing seasons, morphological, physiological, biochemical, and molecular responses were assessed. The data showed that the optimal doses of KFeO_2_-NPs were 50 mg/L for the yellow cultivar and 100 mg/L for the white cultivar, resulting in increases the total chlorophyll content up to 55.1 to 52.2% and 57.8 to 54.1%, carotenoids up to 32.2 to 31.9% and 24.9 to 23.1%, green cob yield up to 47.0 to 46.8% and 33.0 to 33.7%, total phenol up to 37.2 to 38.4% and 28.3–26.1%, and total indoles up to 30.3–30.5% and 32.0 to 31.9%, during two seasons for yellow and white cultivar, respectively. Toxicity assessment confirmed the complete safety of the grains, with EC₅₀ values exceeding 100, ranging from 102 to 125 and 108 to 135 for yellow and white cultivars, respectively. This indicates that they are non-toxic and environmentally safe. At higher KFeO_2_-NPs concentrations (100 mg/L), ISSR analysis showed only slight genomic changes, where the genomic template stability dropped to 82.81% in yellow corn and 75.36% in white corn. All things considered, these results demonstrate that KFeO_2_-NPs function as effective foliar nanofertilizers, enhancing sweet corn yield, growth, and biochemical composition while highlighting the significance of dose optimization to strike a compromise between genomic integrity and productivity gains.

**Supplementary Information:**

The online version contains supplementary material available at 10.1186/s12870-026-08131-7.

## Introduction

Sweet corn (*Zea mays* L. saccharata) is a maize type characterized by a high sugar content and tender kernels cultivated primarily for human consumption. Due to its short growth season and soil-type adaptability, it is commonly grown in both temperate and tropical locations. It was obtained via a naturally occurring recessive mutation in the genes governing the conversion of sugar to starch within the maize kernel’s endosperm [1]. It is harvested when it is still immature (milk stage), cooked, and consumed as a vegetable rather than a grain. It is distinguished by a high content of carbohydrates, dietary fiber, vitamins (including vitamin C and B-complex), and vital minerals like phosphorus and magnesium. In addition, phenolic acid, an anti-cancer compound, is increased when sweet corn is cooked [2]. Sweet corn must be consumed fresh, canned, or frozen before the kernels become tough and starchy because the maturation process turns sugar into starch, which makes it difficult to keep. Along with dent corn, flint corn, pod corn, popcorn, and flour corn, sweet corn is one of the six main varieties of corn [3].

Agricultural fertilizers are the source of required plant nutrients, which are used to promote plant growth and yield. The nutrients are classified into two categories: macronutrients (nitrogen, phosphorus, and potassium) and micronutrients (zinc, iron, copper, calcium, magnesium, and molybdenum) [4]. These fertilizers are often applied by adding them to the soil. To improve agricultural production, it was proposed to increase the amount of additional fertilizer [5]. However, the continued and large amount application of conventional fertilizers has resulted in substantial international environmental challenges [6, 7]. To boost agricultural productivity while reducing environmental pollution, researchers are developing fertilizers and improving the efficiency of plant-nutrient usage. Recently, nanotechnology has been used to increase crops yield and quality via enhancement the nutrient usage efficiency [8].

Nanotechnology is a new emerging technology that is the study of material manipulation at the nanoscale (1–100 nm). It is applied in several scientific domains, including agriculture, where it provides creative ways to boost the productivity, sustainability, and efficiency of agricultural systems in the face of escalating global issues such as soil erosion, climate change, water scarcity, and rising food demand [9]. It can significantly provide various solutions to many issues of agriculture compared to traditional agricultural systems. It is used in a variety of agricultural fields, such as crop yield and quality, post-harvest management, soil improvement, and plant protection [10, 11]. The creation of nano-pesticides and nano-fertilizers, which enable the targeted and controlled release of agrochemicals and nutrients to reduce losses and environmental pollution, is one of the most promising uses [12]. These nano-formulations encourage sustainable agricultural methods, decrease the frequency of applications, and improve the efficiency of nutrient uptake [13]. Furthermore, the way that NPs interacted with plants was influenced by their size, shape, and dosage. Additionally, using nano-fertilizers could enhance existing functions or introduce new ones [14, 15].

An overabundance of nanoparticles disrupts the oxidative process and causes the formation of ROS. The incoming nanoparticles may interact with the electron transport chain of mitochondria and chloroplasts, possibly leading to an oxidative burst that is indicated by an increase in ROS concentration [16–18]. Van Breusegem and Dat [19] state that once ROS is produced due to nanoparticle interaction, it interacts with almost every aspect of the cell, resulting in protein alterations, lipid peroxidation, and DNA damage. Necrosis or apoptosis are two ways that increased ROS production might kill plant cells. DNA fingerprinting is a key molecular marker technique used to detect mutagenic effects resulting from the interaction between chemicals or heavy metals and plants [20]. Molecular markers such as ISSR are effective tools for identifying changes in DNA fingerprints, which reflect genetic variations within the genome [21]. ISSR markers offer several advantages, including simplicity, speed, low cost, and the fact that they do not require prior knowledge of genomic sequences [22]. These DNA-based markers detect polymorphisms between microsatellite regions, and the resulting DNA profiles can vary due to the emergence or loss of bands, or changes in their intensity [23].

Although the effects of nano-fertilizers on plant growth have been the subject of several studies, little is known about the production, physicochemical characterization, and safe foliar application of KFeO_2_-NPs in field settings. In addition, it is challenging to develop trustworthy recommendations for agronomic use since current commercial nano-fertilizers frequently lack thorough documentation of particle size, stability, purity, and dose-dependent plant responses. Furthermore, nothing is known about the possible effects of varying KFeO_2_-NPs concentrations on plant stress tolerance and genomic stability. So, this study aims to synthesize and characterize KFeO_2_-NPs and investigate their impact as a foliar fertilizer on the growth, yield components, and grain quality of the tested cultivars, as well as evaluate their influence on biochemical traits, including antioxidant activity, proline content, pigments, and nutrient accumulation. Additionally, genomic stability under various KFeO_2_-NP concentrations was examined using ISSR markers to detect potential genotoxic effects. Also, the optimal KFeO_2_-NP dose that maximizes agronomic performance without causing physiological or genetic stress was determined to establish safe and effective application levels.

## Materials and methods

Two cultivars of sweet corn seeds, yellow (Jingke sweet 183) and white (JingkeNuo 2000), were brought from China (Beijing AgriaNky seed Co., Ltd). Ferric nitrate, ethylene glycol, and potassium nitrate were purchased from S.D. Fine-Chem.

### Synthesis of potassium ferrite nanoparticles (KFeO_2_-NPs)

KFeO_2_-NPs were synthesized as a source of potassium via the sol-gel method [24]. A potassium nitrate solution (1 M) was mixed with a ferric nitrate solution (2 M), followed by the addition of citric acid (2 M) and ethylene glycol (5 mL). The mixture was continuously stirred and heated at 80–90 °C, leading to the formation of a viscous brown gel. Upon further heating, the gel dried and converted to a brown powder. This powder was washed thoroughly with ethanol and distilled water, then dried overnight in a vacuum oven at 60 °C. To enhance crystallinity and eliminate impurities, it was calcined at 550 °C for 2 h, yielding KFeO_2_-NPs. The morphology was examined using a TEM (JEM-1230, Japan) operated at 120 kV with a resolution of 0.2 nm. The crystal structure was studied using XRD analysis that was performed using a Diano-diffractometer with CoKα radiation (45 kV) and a Philips X-ray diffractometer (PW 1930, PW 1820) with CuKα radiation (λ = 0.15418 nm). The crystallite size was calculated using the Scherrer equation:

$$\:D=\:\:K\lambda\:/\beta\:cos\theta\:$$

Where D is the crystallite size, K is the shape factor (0.9), β is the peak’s full-width at half-maximum (FWHM) in radians, λ is the X-ray wavelength, and θ is the Bragg angle.

### Experiment layout

By the end of February, the soil had been scrubbed, ploughed, leveled, and then divided into plots. During soil preparation, organic manure was applied at a rate of 47.6 m^³^/ha, along with 476 kg/ha of calcium superphosphate (15.5% P₂O₅). Potassium sulphate (119 kg/ha; 48% K₂O) and ammonium nitrate (285.6 kg/ha; 33.5% N) were applied in split doses after seed germination. Sweet corn seeds were sown on 1 st March during the 2023 and 2024 seasons in clay soil located in Shebin El-Kom, El-Menoufia Governorate, Egypt (30° 33’ N, 31° 0’ E, 20 m a.s.l.). The average of the climatic conditions during the two years, including average high and Low temperatures (°C), relative humidity (%), as well as rainfall (mm), are represented in Table 1.

**Table 1:** The average of the climatic conditions during the two years 2023 and 2024

Month	High Temperature (°C)	Low Temperature (°C)	Relative Humidity (%)	Rainfall (mm)
March	~ 24.3	~ 12.5	~ 46%	~ 3 mm
April	~ 28.6	~ 15.3	~ 40%	~ 1 mm
May	~ 32.9	~ 19.3	~ 37%	~ 0 mm

Two seeds were planted per hill, with each plot covering 25.2 m^2^ and consisting of three ridges, each measuring 12 m × 0.7 m. Plants were spaced 20 cm apart on one side of each ridge. The physical and chemical properties of the soil, averaged over the two seasons, were analyzed following the methods of Cottenie et al. [25], as shown in Table 2.

**Table 2:** Chemical and physical properties of soil (combined data of two seasons)

Physical properties										
Texture			Clay (%)		Silt (%)		Sand (%)			
Clay			50.4		37.8		11.8			

Chemical properties										
**EC (dS/m)**	**pH**	**mg/L**								
		**N**	**P**	**K**	**Ca**	**Mg**	**Na**	**SO**_**4**_	**CL**	**HCO**_**3**_
0.45	7.47	3.39	26.9	384	43.68	8.02	25.97	160.9	41.12	84.21

### Experiment treatments

The yellow and white sweet corn cultivars were foliar-sprayed with KFeO_2_-NPs at different concentrations (0, 25, 50, 75, and 100 mg/L) after 25 days of sowing (Vegetative Growth Stages; V4). A second spray was applied 15 days after the first. The experiment followed a split-plot design with three replications. The main plots were assigned to the cultivar types, while the KFeO_2_-NPs concentrations were randomly distributed within the sub-plots.

### Photosynthetic pigments

Photosynthetic pigments were analyzed from fresh leaf samples collected 50 days after sowing. They were performed on the fourth leaf of the plant, using extraction with 85% acetone as described by Vaccari et al. [26]. The concentrations of chlorophyll a (Chl. a), chlorophyll b (Chl. b), and carotenoids (Cart) were determined using a UV/VIS spectrophotometer (TG 80, Germany) at wavelengths of 663, 644, and 452 nm, respectively, with 85% acetone serving as the blank, according to Metzner et al. [27]. Pigment concentrations were initially expressed in µg/mL and calculated using formulas proposed by Jiang et al. [28], then converted to mg/g of fresh plant material.

### Agro-morphological criteria

Three plants were randomly selected from each treatment after 70 days of sowing (Silk stage; R1) to assess vegetative growth parameters, including plant length (cm), leaf area (cm^2^), number of leaves, number of internodes, and fresh weight of both stem and leaves (g). At the harvest stage (90 days after sowing), measurements were taken for cob weight (g), diameter (cm) and length (cm), number of rows per cob, number of grains per row, and yield (t/ha).

### Biochemical contents

Fresh sweet corn grain samples were oven-dried at 60 °C until a constant weight was achieved, then ground into a fine powder. The extract was then prepared using 80% ethanol for subsequent analyses.

#### Mineral determination

To determine nitrogen (N), phosphorus (P), and potassium (K) levels, 0.5 g of dried grain sample was digested in a flask with 10 mL of concentrated sulfuric acid (H₂SO₄). After heating for 10 min, 1 mL of perchloric acid was added, and the mixture was further heated until a clear solution was obtained. The digest was then diluted to 100 mL with distilled water, following the method of Cottenie et al. [25].

Nitrogen content was analyzed using the Kjeldahl method as described by Khanzada et al. [29]. Following digestion, a 33% sodium hydroxide (NaOH) solution was added to the mixture, which was then subjected to steam distillation. The distillate was captured in 20 mL of a 4% boric acid solution. Nitrogen content was subsequently quantified via titration using 0.01 N hydrochloric acid (HCl).

Phosphorus content in the digested samples was analyzed using the ascorbic acid method outlined by as outlined by Cottenie et al. [25]. For the preparation of the reagent, 125 ml of 5 N sulfuric acid was combined with 37.5 ml of 4% ammonium molybdate solution, followed by the addition of 25 ml of 0.1 M ascorbic acid solution and 12.5 ml of 0.274% potassium antimonyl tartrate solution. This reagent was freshly prepared prior to use. A 1 ml aliquot of the digested solution was transferred into a 50 ml volumetric flask, mixed with 20 ml of the prepared reagent, and then diluted to the calibration mark with distilled water. The optical density of the resulting solution was measured using a spectrophotometer at a wavelength of 620 nm. The phosphorus concentration was determined from a standard curve created using varying concentrations of standard potassium dihydrogen phosphate solutions.

Potassium content was quantified using a flame photometer in the digested samples following the procedure of Okalebo et al. [30]. After the samples have been digested, make up the volume to 100 ml using distilled water. A standard potassium solution of 1000 ppm is prepared, and 10 ml of this solution is transferred to a 100 ml standard flask, to bring the volume to 100 ml of potassium solution. A flame photometer is calibration by feeding the standers. Potassium concentration is expressed as a percentage 100 ml standard flask.

#### Total sugar

The total sugar content was determined following the method described by Dubois et al. (1956). In this procedure, 1 mL of the ethanol extract was mixed with 5% phenol (1 mL) and concentrated sulfuric acid (5 mL) in a test tube. The mixture was gently stirred and left to cool. A blank was prepared using 1 mL of 80% ethanol along with the same reagents. Absorbance was measured at 490 nm using a spectrophotometer.

#### Total phenols

For the phenolic content analysis, 1 mL of the extract was mixed with 70 mL of distilled water, followed by the addition of Folin-Ciocalteu reagent and 15 mL of saturated sodium carbonate solution. The mixture was incubated at room temperature for 30 min, and absorbance was measured at 765 nm using a spectrophotometer. A calibration curve was prepared using gallic acid (GA), following the method of Stratil et al. [31].

#### Total indoles

For total indole content determination, 1 mL of the ethanol extract was added to a test tube, followed by 2 mL of Salkowski reagent (a mixture of concentrated H₂SO₄ (150 mL) and 0.5 M FeCl₃·6 H₂O (7.5 mL), as described by Gordon and Weber [32]. The mixture was incubated in the dark at room temperature for 30 min. The absorbance was then measured at 530 nm using a spectrophotometer, according to Lwin et al. [33]. The concentration of total indoles was calculated using a standard curve prepared with indole acetic acid (IAA).

#### Total flavonoids

The total flavonoid content was determined using the colorimetric method outlined by Chang et al. [34]. To estimate flavonoids, 0.5 mL of the ethanol extract was mixed with 0.1 mL of aluminum chloride (10%), 0.1 mL of potassium acetate (1 M), and 4.3 mL of distilled water. The mixture was incubated at room temperature for 30 min, and the absorbance was measured at 415 nm using a spectrophotometer. Results were expressed as milligrams of quercetin equivalent (QCE) per gram of dry weight.

#### Oil content

The oil content was measured according to the Association of Official Analytical Chemists [35]. Dry grain (5 g; previously dried in an oven at 102 °C till constant weight) was placed into a thimble, which was then inserted into a beaker within a Soxhlet apparatus. A volume of 90 mL of petroleum ether (boiling range 40–60 °C) was added to the flask, which was then heated until the solvent began to boil. The extraction process was carried out over 6 h. Afterward, the thimble was dried in an oven at 102 °C for 2 h until a constant weight was reached. The oil content was calculated using the following equation:

$$oil\:\%=\:\frac{{w}_{2}-{w}_{1}}{s}\:X\:100$$

Where the weight of the empty thimble (g) is W_1_, the weight of the thimble plus sample (g) is W_2,_ and the weight of the sample is S.

#### Total protein

A portion of the dried and ground grain samples (0.5 g) was placed into a digestion flask and digested using 10 mL of concentrated sulfuric acid (H₂SO₄). After digestion, 33% sodium hydroxide (NaOH) was added, and the mixture underwent steam distillation. The resulting distillate was collected in 20 mL of 4% boric acid solution. The nitrogen content was then determined through titration with 0.01 N hydrochloric acid (HCl). Total protein content was calculated by multiplying the measured nitrogen percentage by a factor of 6.25 [29].

### Statistical analysis

The collected data were analyzed using the Two-way analysis of variance ANOVA in SPSS Statistical software for the Social Sciences, version 17.0 for Windows (SPSS Inc., Chicago, IL, USA, 2008 release). The results are expressed as the mean of three replicates ± standard deviation (SD). To determine the statistically significant difference among treatment means, the least significant differences (LSD) test was applied at a significance level of *p* ≤ 0.05, following the methodology described by Snedecor and Cochran [36].

### Toxicity test

Aqueous extracts were prepared from the dried and ground sweet corn seeds at two concentrations: 0.1% and 1.0%. The toxicity of these extracts was evaluated using a Microtox 500 analyzer (USA), following the procedure outlined by Johnson [37]. This analysis was performed on samples from the second season 2024.

### DNA extraction and Inter-simple sequence repeat - polymerase chain reaction (ISSR-PCR) amplification

Randomly selected leaf samples from shoots of each treatment were used to extract genomic DNA using the GeneJET Plant Genomic DNA Purification Mini Kit (Thermo Scientific, K0791). The quantity of the extracted DNA was measured using a NanoDrop 1000 spectrophotometer (Thermo Scientific) and adjusted to a concentration of 50 ng/µL for use as a template in PCR reactions.

Eight ISSR primers were selected according to Hou et al. [38]. These primers were employed for PCR amplification of the genomic DNA from both white and yellow corn (Table 3). PCR was carried out in 0.2 mL Eppendorf tubes with a final reaction volume of 25 µL in 96-well plates, using a Bio-Rad Thermocycler. Each PCR mixture contained 12.5 µL of Dream Taq Green PCR Master Mix 2X (Thermo Scientific, K1081), 1 µL of 10 pmol primer (Metabion, Germany), 1 µL of template DNA (50 ng/µL), and nuclease-free water to reach a total volume of 25 µL. The PCR cycling conditions included an initial denaturation at 94 °C for 1 min, followed by 35 cycles of denaturation at 94 °C for 1 min, annealing at 55 °C for 30 s, and extension at 72 °C for 1 min. A final extension step was performed at 72 °C for 5 min, followed by a hold at 4 °C. For electrophoresis, 5 µL of each amplified product and DNA ladder 100 bp (GeneDirex, Cat No. DM003-R500) were loaded into wells of a 1.5% agarose gel prepared with 1X TAE buffer and run at 100 V for approximately 2 h. Ethidium bromide (0.5 g/ml) was used to make bands visible under UV light by using a Bio-Rad gel documentation apparatus. Data analysis: ISSR data were scored for presence (1), absence (0), and analyzed using the Total Lab program to detect the molecular size of each band.

The Genome Templet Stability percentage (GTS%) was computed as follows:

$$GTS{\%}=100-(100a/n)$$

Where (a) represents the average number of differences in DNA profiles, and (n) represents the number of bands picked from DNA control profiles [39].

Also, the Relative migration distance (Rf) was calculated by the following equation [40].

$$Rf=(distance\:traveled\:by\:protein\:band)/(distance\:traveled\:by\:dye\:front)$$

**Table 3:** Sequences of eight ISSR primers

Ser. No.	ISSR Primers	Primer sequence (5′−3′)
1	ISSR-2	(AG)8 C
2	ISSR-3	(GA)8T
3	ISSR-4	(CT)8G
4	ISSR-5	(CA)6ACAG
5	ISSR-6	BDB(TCC)5
6	ISSR-7	HVH(TCC)5
7	ISSR-8	DBDA(CA)7
8	ISSR-12	(AG)8YT

^*^Y = G/C, B = T/G/C; D = A/T/G, H = A/T/C, V = A/G/C

## Results

### Characterization of KFeO_2_-NPs

**Fig. 1:**
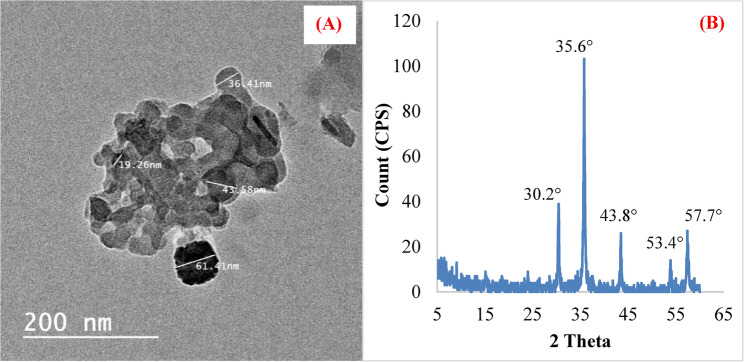
TEM (**A**) and XRD pattern (**B**) of KFeO_2_-NPs fertilizer

Figure 1A and B display the morphological characteristics and crystal structure of the synthesized KFeO_2_-NPs. Transmission electron microscopy revealed that the synthesized KFeO_2_-NPs had an average particle size of 45 nm ± 10 nm (Fig. 1A**)**. The X-ray diffraction analysis of KFeO_2_-NPs showed prominent diffraction peaks at 2θ values of 30.2°, 35.5°, 43.8°, 53.4°, and 57.6° corresponding to the planes (131), (220), (117), (236), and (324), respectively, which are consistent with the orthorhombic crystal structure of KFeO_2_-NPs (Fig. 1B**)**. These results confirm the successful synthesis and structural integrity of the prepared nanomaterials. The crystal size was equal to 23.1 nm, calculated by using the Scherrer equation.

### Photosynthetic pigments

**Fig. 2:**
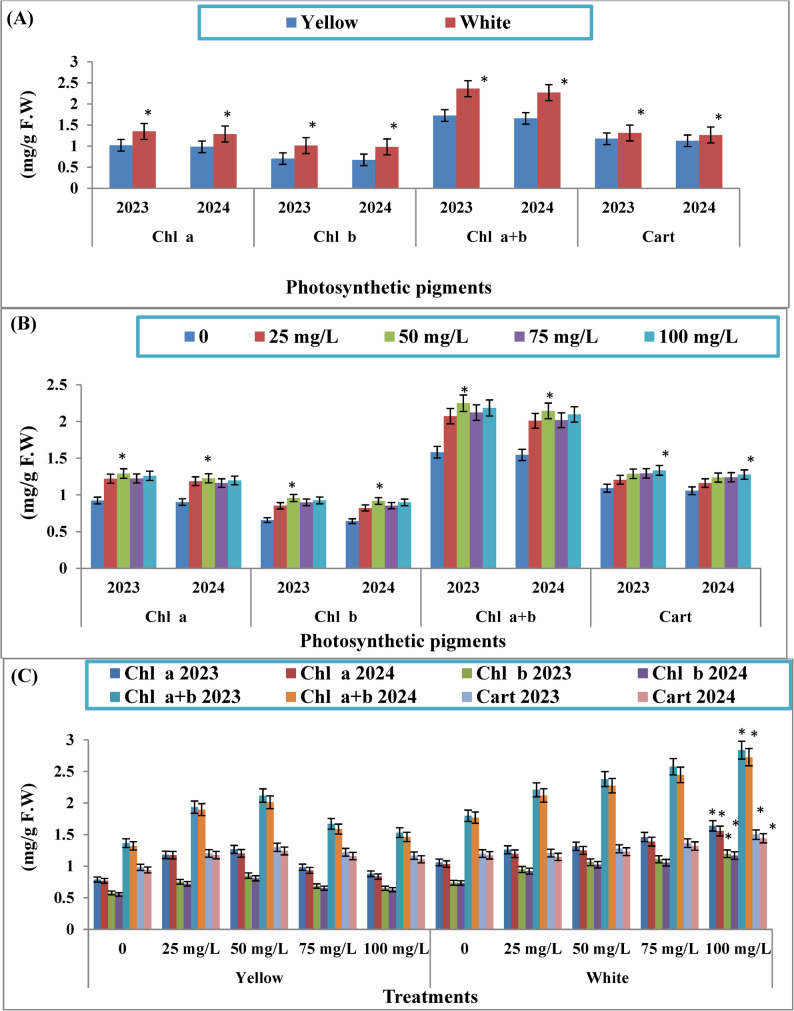
Effect of cultivars (**A**), different concentrations of KFeO_2_-NPs (**B**) and their interaction (**C**) on photosynthetic pigments of sweet corn plant during the two following planting seasons. The data are presented as means ± stander deviation (*n* = 3). The asterisk (*) indicates a significant difference between treatments at a significance level of *p* < 0.05 according to the LSD test

The sweet corn cultivars had a significant effect on photosynthetic pigment content, including Chl a, Chl b, total Chl, and Cart, during the 2023 and 2024 growing seasons (Fig. 2A**)**. The white cultivar consistently exhibited the highest levels of all measured pigments compared to the yellow cultivar. The increase in Chl a, Chl b, total Chl, and Cart in the leaves of white sweet was (32.16 and 30.69%), (43.75 and 45.77%), (36.81 and 36.87%), and (11.57 and 12.17%), respectively, during the two study seasons compared with yellow sweet corn.

Regarding KFeO_2_-NPs treatments, foliar application at 50 mg/L significantly increased the content of Chl a (60.4–56.5), Chl b (47.2–46.2), total Chl (55.1–52.2%), and Cart (32.2–31.9%) in yellow sweet corn leaves compared to the control during two seasons, respectively. Conversely, the foliar application at 100 mg/L significantly increased the content of Chl a (45.7–49.7), Chl b (62.3–59.2), total Chl (57.8–54.1%), and Cart (24.9 to 23.1%) in white sweet corn leaves compared to the control during two seasons, respectively (Fig. 2B).

Similarly, the interaction between cultivar type and KFeO_2_-NPs concentration had a significant impact at level significant 5% on all measured photosynthetic pigments. The combination of the white cultivar and 100 mg/L of KFeO_2_-NPs resulted in the highest levels of Chl a, Chl b, total Chl, and Cart during both the 2023 and 2024 seasons (Fig. 2C).

### Vegetative growth characters

Mean vegetative growth characteristics of sweet corn cultivars treated with varying concentrations of KFeO_2_-NPs during the 2023 and 2024 seasons are presented in Table 4. The white sweet corn cultivar showed statistically significant improvements in all measured vegetative traits, including plant height, leaf area, number of leaves and internodes, and the fresh weight of leaves and stems by about (34.4 and 34.7%), (25.4 and 25.4%), (43.4,51.4), (28.0 and 27.0%), (73.3 and 73.3%), and (97.0 and 97.1%), respectively, during two sequential seasons 2023 and 2024 as compared to yellow sweet corn.

**Table 4:** Effect of cultivars, different concentrations of KFeO_2_-NPs and their interaction on the vegetative characteristics of sweet corn plant

Cultivars	KFeO_2_-NPs concentrations) mg/L(	Plant length(cm)			Leaf area(cm^2^)		No.				Fresh weight (g)				
							Leaves		Internodes		Leaves		Stem		
		2023		2024	2023	2024	2023	2024	2023	2024	2023	2024		2023	2024
Yellow	Control	140.5±0.46		133.5±0.44	439.2±0.45	417.3±0.43	7.0±0.58	6.8±0.20	8.0±1.00	7.6±0.05	64.7±4.23	61.6±1.00		152.3±4.75	144.7±4.52
	25	150.2±0.20		142.7±0.19	425.0±5.70	403.8±5.41	7.0±0.50	6.7±0.47	9.3±0.58	8.9±0.55	90.2±2.11	86.1±1.41		381.0±2.00	361.9±1.90
	50	148.0±2.00		140.6±1.90	551.1±6.05	523.5±5.75	8.0±1.00	7.6±0.95	10.0±1.00	9.5±0.50	92.3±2.30	87.7±2.19		401.4±1.40	381.3±1.33
	75	143.0±3.00		135.9±2.85	506.8±3.30	481.5±3.13	8.1±0.85	7.9±0.54	9.5±0.50	9.4±0.36	86.8±3.20	82.5±3.04		461.1±4.01	438.1±3.81
	100	142.7±0.70		135.5±0.67	482.6±3.15	458.5±2.99	7.7±0.58	7.2±0.20	9.5±0.50	9.0±0.47	74.4±4.21	70.9±3.64		383.5±1.04	364.3±1.38
Mean			**144.9**	**137.6**	**480.9**	**456.9**	**7.6**	**7.2**	**9.3**	**8.9**	**81.7**	**77.8**		**355.9**	**338.1**
White	Control	203.0±3.00		194.9±1.74	533.6±4.95	506.9±4.70	10.3±0.58	9.8±0.10	12.3±0.61	11.7±0.58	130.0±5.00	123.5±4.75		555.0±5.00	529.6±2.51
	25	209.0±6.56		198.6±6.23	557.5±0.06	529.7±0.05	9.4±0.38	9.4±0.10	11.0±1.00	10.5±0.05	128.3±5.69	122.6±1.66		623.3±7.64	592.2±7.26
	50	196.0±4.00		186.2±3.80	586.7±0.75	557.4±0.71	11.0±1.00	10.5±0.95	11.7±0.58	11.1±0.55	146.7±4.73	139.3±3.05		700.0±5.00	666.0±2.65
	75	189.0±1.00		179.6±0.95	643.1±5.55	610.9±5.27	11.3±1.15	10.7±0.61	12.0±1.00	11.4±0.05	148.3±4.73	140.9±4.49		758.3±9.36	723.4±5.72
	100	176,3±3.91		167.5±3.72	695.3±2.30	660.5±2.18	12.3±0.58	11.9±0.10	12.3±0.58	11.7±0.55	154.9±4.20	147.6±4.33		863.7±7.09	820.5±6.74
Mean			**194.7**	**185.3**	**603.2**	**573.1**	**10.9**	**10.9**	**11.9**	**11.3**	**141.6**	**134.8**		**700.1**	**666.3**
Mean	Control	171.8		164.2	486.4	462.1	9.0	8.3	10.2	9.7	97.4	92.6		353.6	337.1
	25	179.6		170.6	491.3	466.7	8.2	8.0	10.2	9.7	109.3	104.3		502.2	477.0
	50	172.0		163.4	568.9	540.5	9.5	9.0	10.8	10.3	119.5	113.5		550.7	523.6
	75	166.0		157.7	574.9	546.2	9.7	9.3	10.8	10.4	117.6	111.7		609.7	580.7
	100	159.5		151.5	589.0	559.5	10.0	9.5	10.9	10.4	114.6	109.3		623.6	592.4
LSD 5%	Cultivars	**9.97**		**10.92**	**5.49**	**5.21**	**0.96**	**0.71**	**0.63**	**1.51**	**5.17**	**3.58**		**109.5**	**104.47**
	KFeO_2_-NPs concentrations	**10.06**		**9.39**	**4.92**	**4.67**	**0.73**	**0.54**	**N.S.**	**N.S.**	**9.28**	**8.68**		**53.4**	**50.59**
	Interaction	**14.23**		**13.28**	**6.95**	**6.60**	**1.04**	**0.76**	**1.31**	**1.26**	**13.12**	**12.27**		**75.6**	**71.55**

*N.S*. Not Significant (p< 0.05)

The foliar application of KFeO_2_-NPs at 100 mg/L resulted in the greatest values for leaf area (21.1 and 21.1%), number of leaves (11.1 and 14.45%), internodes (6.9 and 7.2%), and stem fresh weight (76.4 and 75.7%), compared with control, during two agriculture seasons 2023 and 2024. Interestingly, the longest plants were observed at the lowest concentration (25 mg/L) by about (4.5 and 3.9%) compared with control, while leaf fresh weight was also notably enhanced with the 50 mg/L treatment by about (22.7 and 22.6%) (Table 4).

Furthermore, the interaction between sweet corn cultivars and KFeO_2_-NPs concentrations had a significant effect on most vegetative growth parameters, namely plant height, leaf area, number of leaves, and fresh weight of leaves and stems across both seasons (Table 4). However, the number of internodes was not significantly affected by the interaction.

### Yield characters

The cob characterization of sweet corn cultivars treated with varying concentrations of KFeO_2_-NPs over the 2023 and 2024 growing seasons is shown in Table 5. The white cultivar showed significantly superior cob traits, including cob length, diameter, number of grains per row, and green cob yield compared to the yellow cultivar. The percentage increase observed for white corn, compared to yellow corn, was evident in the number of grains per row (51.9% and 51.9%), cob weight (51.9% and 51.9%), and yield (52.7% and 52.9%), during two consecutive growing seasons. However, the yellow cultivar exhibited a higher number of rows per cob across both seasons.

**Table 5:** Effect of cultivars, different concentrations of KFeO_2_-NPs and their interaction on the yield of sweet corn plant

Cultivars	KFeO_2_-NPs concentrations ) mg/L(	Cob length (cm)		Cob diameter (cm)		No.				Cob weight (g)		Green cob yield (t/h)	
						Rows/cob		Grains/row					
		2023	2024	2023	2024	2023	2024	2023	2024	2023	2024	2023	2024
Yellow	Control	17.7 ± 0.58	16.8 ± 0.55	4.80 ± 0.20	4.56 ± 0.19	13.0 ± 1.00	12.4 ± 0.05	30.7 ± 1.13	29.1 ± 0.90	165.8 ± 4.20	157.5 ± 3.99	23.6 ± 0.24	22.4 ± 0.23
	25	19.7 ± 0.29	19.7 ± 0.25	5.03 ± 0.06	4.78 ± 0.05	14.0 ± 0.50	13.3 ± 0.30	35.0 ± 1.73	32.6 ± 1.46	180.0 ± 5.00	171.0 ± 4.75	25.7 ± 0.48	24.4 ± 0.45
	50	19.2 ± 0.76	18.4 ± 0.56	5.07 ± 0.35	4.81 ± 0.33	16.0 ± 1.00	15.2 ± 0.06	36.3 ± 0.89	34.5 ± 0.84	247.0 ± 3.00	234.7 ± 2.85	34.7 ± 1.07	32.9 ± 1.02
	75	18.7 ± 0.35	18.2 ± 0.25	5.00 ± 0.10	4.90 ± 0.10	14.7 ± 0.30	13.9 ± 0.18	35.7 ± 0.58	33.9 ± 0.55	186.9 ± 3.10	177.5 ± 2.95	26.9 ± 0.71	25.5 ± 0.68
	100	18.3 ± 0.30	18.0 ± 1.00	4.97 ± 0.28	4.84 ± 0.17	13.3 ± 0.30	12.7 ± 0.18	34.3 ± 0.65	33.3 ± 1.70	179.9 ± 2.05	170.9 ± 1.95	25.7 ± 0.48	24.4 ± 0.45
Mean		18.7	**18.2**	**4.97**	**4.78**	**14.2**	**13.5**	**34.4**	**32.7**	**191.9**	**182.3**	**27.3**	**25.9**
White	Control	21.8 ± 0.20	20.4 ± 0.311	4.93 ± 0.15	4.69 ± 0.15	11.3 ± 0.58	10.8 ± 0.29	36.0 ± 1.00	34.2 ± 0.95	241.7 ± 5.77	229.6 ± 5.48	34.5 ± 1.19	32.8 ± 1.13
	25	20.8 ± 0.58	19.5 ± 0.50	4.97 ± 0.15	4.72 ± 0.15	12.0 ± 1.00	11.4 ± 0.06	38.3 ± 1.15	36.4 ± 1.10	285.0 ± 5.00	270.8 ± 4.75	40.7 ± 0.24	38.7 ± 0.23
	50	21.1 ± 1.00	20.8 ± 0.36	5.03 ± 0.15	4.83 ± 0.10	12.7 ± 1.15	12.0 ± 1.10	41.0 ± 2.65	38.0 ± 1.90	297.0 ± 6.08	282.2 ± 5.78	42.4 ± 0.48	40.2 ± 0.45
	75	22.4 ± 0.36	21.3 ± 0.34	5.13 ± 0.13	4.90 ± 0.10	13.3 ± 1.15	12.7 ± 1.10	42.0 ± 2.65	39.9 ± 2.51	306.7 ± 7.64	291.3 ± 2.11	45.0 ± 0.24	42.7 ± 0.23
	100	23.3 ± 0.29	22.2 ± 0.20	5.23 ± 0.06	5.07 ± 0.03	13.7 ± 0.58	13.0 ± 0.30	47.0 ± 3.00	44.7 ± 2.85	327.0 ± 2.60	310.7 ± 2.51	45.9 ± 0.71	43.6 ± 0.68
Mean		**21.8**	**20.8**	**5.06**	**4.84**	**12.6**	**12.0**	**40.9**	**38.6**	**291.5**	**276.9**	**41.7**	**39.6**
Mean	Control	19.7	18.6	4.87	4.62	12.2	11.6	33.3	31.7	203.7	193.5	29.0	27.6
	25	20.1	19.6	5.00	4.75	13.0	12.4	36.7	34.5	232.5	220.9	33.2	31.5
	50	20.2	19.6	5.05	4.82	14.3	13.6	38.7	36.3	272.0	258.4	38.5	36.6
	75	20.5	19.8	5.07	4.90	14.0	13.3	38.8	36.9	246.8	234.4	35.9	34.1
	100	20.8	20.1	5.10	4.95	13.5	12.8	40.7	39.0	253.4	240.8	35.8	34.0
LSD 5%	Cultivars	**0.75**	**0.27**	**N.S.**	**N.S.**	**1.31**	**1.25**	**2.23**	**3.10**	**35.04**	**33.29**	**0.76**	**0.72**
	KFeO_2_-NPs concentrations	**0.71**	**0.70**	**0.03**	**0.04**	**1.13**	**1.07**	**2.16**	**3.84**	**24.64**	**23.40**	**0.71**	**0.68**
	Interaction	**1.01**	**0.99**	**N.S.**	**N.S.**	**1.59**	**1.51**	**3.06**	**N.S.**	**34.84**	**33.10**	**1.01**	**0.96**

*N.S*. Not Significant (*p* < 0.05)

The application of KFeO_2_-NPs with 50 mg/L resulted in notable increases in the number of rows per cob (17.2 and 14.7%), cob weight (33.5 and 33.5%), and green cob yield (32.8 and 32.6%), compared to the control, during both years. In contrast, the highest cob length (5.6 and 8.1%), cob diameter (4.7 and 7.1%), and number of grains per row (32.6 and 23.0%) were observed with the 100 mg/L treatment compared to the control. All yield-related traits showed significant effects with varying concentrations of KFeO_2_-NPs, with the exception of the cob diameter. All yield-related traits showed significant effects with varying concentrations of KFeO_2_-NPs, except for cob diameter.

The interaction between sweet corn cultivars and KFeO_2_-NPsconcentrations was statistically significant across both seasons (Table 5). The white cultivar treated with 100 mg/L KFeO_2_-NPs demonstrated the greatest improvements in cob length, number of rows per cob, number of grains per row, cob weight, and green cob yield. Conversely, the cob diameter of rows per cob did not show a significant increase, during the 2023 and 2024seasons.

N.S. = Not Significant (*p* < 0.05).

### Minerals

**Fig. 3:**
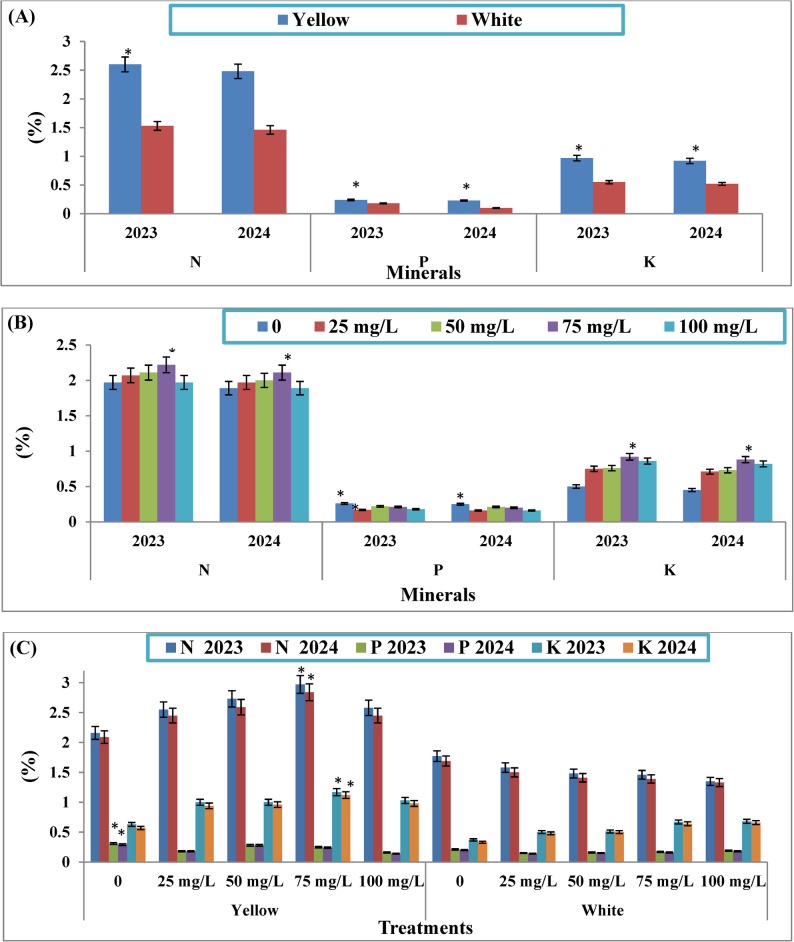
Effect of cultivars (**A**), different concentrations of KFeO_2_-NPs (**B**) and their interaction (**C**) on minerals of sweet corn grains during the two consecutive planting seasons, 2023 and 2024. The data are presented as means ± stander deviation (*n* = 3). The asterisk (*) indicates a significant difference between treatments at a significance level of *p* < 0.05 according to the LSD test

The data showed that the mineral content of sweet corn grains was significantly influenced by cultivar type during the 2023 and 2024 seasons (Fig. 3**)**. Grains from the yellow cultivar exhibited higher levels of nitrogen (2.60 and 2.48%), phosphorus (0.24 and 0.23%), and potassium (0.97 and 0.92%), respectively, during two planting seasons as compared to those from the white cultivar.

Regarding the effect of KFeO_2_-NPs treatments, the highest concentrations of nitrogen (2.22 and 2.11%) and potassium (0.92 and 0.88%) in grains were observed with 75 mg/L of KFeO_2_-NPs, which was significantly superior to other concentrations in both seasons. In contrast, the highest phosphorus content was recorded under traditional agricultural practices (0.26 and 0.25%), followed by treatment with 50 mg/L of KFeO_2_-NPs (0.22 and 0.21%), respectively, during two consecutive growing seasons.

The interaction between cultivar type and KFeO_2_-NPs concentration had a statistically significant effect (at the 5% level) on mineral content in sweet corn grains across both years. The yellow cultivar treated with 75 mg/L of KFeO_2_-NPs achieved the maximum nitrogen (2.97 and 2. 84%) and potassium content (1.17 and 1.12%) on a dry weight basis, respectively, during two growing seasons 2023 and 2024. However, the highest phosphorus concentration (0.31 and 0.29%) was found in grains of the yellow cultivar grown under conventional conditions.

### Biochemical contents

The results presented that all measured biochemical constituents in sweet corn grains, namely total sugars, oil, protein, total phenols, total flavonoids, and total indoles, were significantly influenced by cultivar during the 2023 and 2024 seasons (Table 6). The white cultivar recorded higher levels of total sugars, oil, and total indoles, whereas the yellow cultivar exhibited significantly greater protein, total phenol, and total flavonoid contents.

In terms of KFeO_2_-NPs treatments, different concentrations had a significant effect on all biochemical traits in sweet corn grains. The application of 25 mg/L KFeO_2_-NPs led to the highest levels of total sugars and total flavonoids, while 50 mg/L resulted in the greatest increase in total phenols. The concentration of 75 mg/L was most effective in enhancing protein and total indole contents. Conversely, the highest oil content was observed at the 100 mg/L concentration.

Additionally, a significant interaction was observed between sweet corn cultivars and KFeO_2_-NPs concentrations for most biochemical parameters, specifically total sugars, protein, total phenols, total flavonoids, and total indoles during both growing seasons (Table 6). However, oil content did not exhibit a statistically significant change in response to this interaction.

**Table 6:** Effect of cultivars, different concentrations of KFeO_2_-NPs and their interaction on biochemical contents of sweet corn plant

Cultivars	KFeO_2_-NPs concentrations ) mg/L(	Total sugar (%)		Oil (%)		Protein (%)		Total phenols (mg GAE/g DW)		Total flavonoids (mg QCE/g DW)		Total Indoles (mg IAA/g DW)	
		2023	2024	2023	2024	2023	2024	2023	2024	2023	2024	2023	2024
Yellow	Control	41.6 ± 2.23	40.4 ± 0.78	14.5 ± 0.10	14.0 ± 0.30	13.5 ± 0.33	12.6 ± 0.10	10.93 ± 0.08	10.32 ± 0.03	2.11 ± 0.17	2.01 ± 0.16	10.03 ± 0.55	9.66 ± 0.13
	25	59.0 ± 1.00	57.0 ± 2.95	15.4 ± 0.66	15.3 ± 1.06	15.9 ± 0.31	14.5 ± 0.17	14.13 ± 0.15	13.49 ± 0.15	3.47 ± 0.06	3.42 ± 0.08	10.90 ± 0.36	10.36 ± 0.34
	50	54.7 ± 0.12	52.6 ± 1.60	16.3 ± 0.81	15.5 ± 1.00	17.1 ± 0.32	15.4 ± 0.29	15.0 ± 0.40	14.29 ± 0.32	3.20 ± 0.20	3.17 ± 0.05	13.07 ± 0.25	12.61 ± 0.39
	75	45.7 ± 0.20	43.4 ± 2.82	15.9 ± 0.26	14.9 ± 0.10	18.6 ± 0.19	17.0 ± 0.17	13.27 ± 0.40	12.69 ± 0.23	2.93 ± 0.07	2.88 ± 0.13	12.37 ± 0.40	11.95 ± 0.11
	100	34.5 ± 2.50	33.3 ± 1.70	15.2 ± 1.06	14.5 ± 0.50	16.1 ± 0.13	14.7 ± 0.11	12.97 ± 0.38	12.37 ± 0.08	2.44 ± 0.10	2.39 ± 0.07	11.24 ± 0.40	10.72 ± 0.11
Mean		**47.1**	**45.3**	**15.5**	**14.8**	**16.2**	**14.8**	**13.26**	**12.63**	**2.83**	**2.77**	**11.52**	**11.06**
White	Control	53.5 ± 2.52	50.8 ± 0.80	25.9 ± 0.38	24.6 ± 0.38	11.1 ± 0.10	10.2 ± 0.10	10.13 ± 0.02	9.86 ± 0.06	1.77 ± 0.22	1.72 ± 0.08	10.83 ± 0.29	10.30 ± 0.26
	25	86.6 ± 4.00	82.3 ± 2.30	26.1 ± 0.40	24.9 ± 0.40	9.9 ± 0.10	8.9 ± 0.15	11.13 ± 0.60	10.84 ± 0.16	2.07 ± 0.29	2.00 ± 0.05	11.80 ± 0.75	11.21 ± 0.72
	50	75.4 ± 5.00	72.7 ± 1.70	26.5 ± 0.23	25.2 ± 0.22	9.3 ± 0.10	8.5 ± 0.21	11.83 ± 0.21	11.40 ± 0.10	2.31 ± 0.22	2.19 ± 0.21	12.80 ± 0.10	12.16 ± 0.09
	75	74.6 ± 0.20	70.9 ± 0.10	27.0 ± 1.42	25.6 ± 1.26	9.1 ± 0.10	8.2 ± 0.07	12.14 ± 0.22	11.86 ± 0.06	2.47 ± 0.32	2.34 ± 0.31	13.57 ± 0.21	12.94 ± 0.14
	100	62.4 ± 2.40	59.3 ± 1.30	27.4 ± 1.19	26.0 ± 1.13	8.5 ± 0.50	8.0 ± 0.10	13.00 ± 0.20	12.44 ± 0.10	2.62 ± 0.36	2.60 ± 0.18	14.30 ± 0.46	13.59 ± 0.44
Mean		**70.5**	**67.2**	**26.6**	**25.3**	**9.6**	**8.8**	**11.65**	**11.28**	**2.2**	**2.17**	**12.66**	**12.04**
Mean	Control	47.6	45.6	20.2	19.3	12.3	11.4	10.53	10.09	1.94	1.86	10.43	9.98
	25	72.8	69.6	20.8	20.0	12.9	11.7	12.63	12.16	2.77	2.71	11.35	10.79
	50	65.0	62.7	21.4	20.4	13.2	11.9	13.42	12.85	2.75	2.68	12.93	12.38
	75	60.2	57.1	21.5	20.2	13.8	12.6	12.71	12.27	2.70	2.61	12.97	12.44
	100	48.5	46.3	21.3	20.2	12.3	11.4	12.98	12.40	2.53	2.49	12.77	12.15
LSD 5%	Cultivars	**7.31**	**6.28**	**0.87**	**0.38**	**0.73**	**0.34**	**0.70**	**0.42**	**0.34**	**0.27**	**0.43**	**0.23**
	KFeO_2_-NPs concentrations	**3.53**	**2.86**	**0.92**	**0.68**	**0.32**	**0.34**	**0.61**	**0.36**	**0.30**	**0.24**	**0.54**	**0.46**
	Interaction	**4.99**	**4.05**	**N.S.**	**N.S.**	**0.45**	**0.48**	**0.87**	**0.51**	**0.42**	**0.34**	**0.76**	**0.65**

*N.S*. Not Significant (*p* < 0.05)

### Toxicity test

**Fig. 4:**
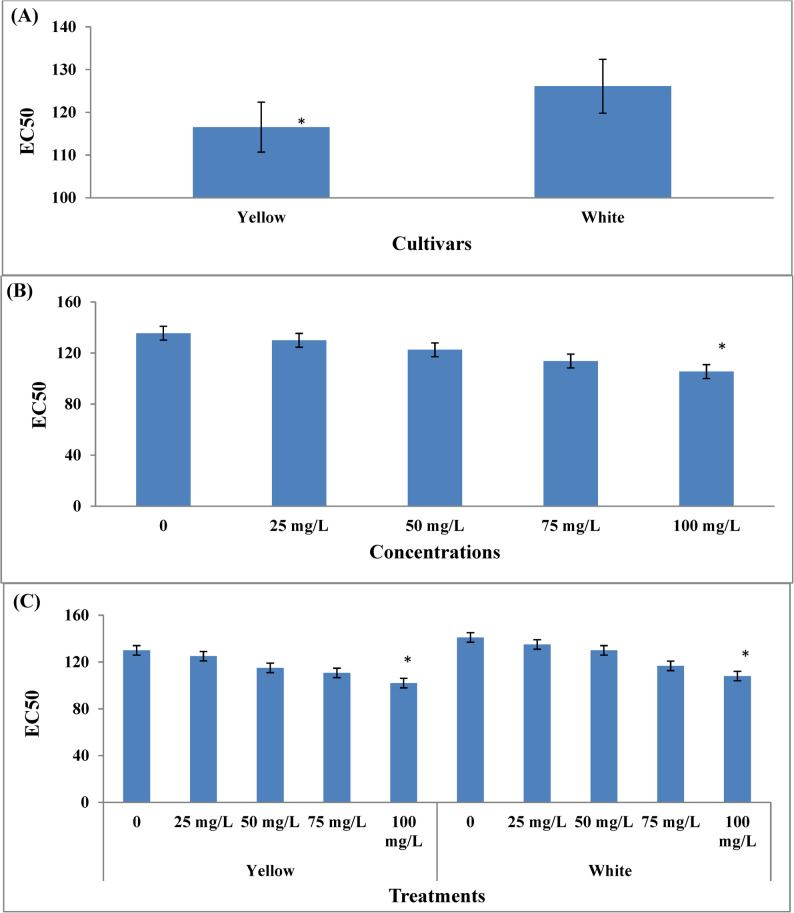
Effect of cultivars (**A**), different concentrations of KFeO_2_-NPs (**B**) and their interaction (**C**) on toxicity in sweet corn grains during growing season 2024. The data are presented as means ± stander deviation (*n* = 3). The asterisk (*) indicates a significant difference between treatments at a significance level of *p* < 0.05 according to the LSD test

Grains of both yellow and white sweet corn, harvested from plants treated with various concentrations of KFeO_2_-NPs (0, 25, 50, 75, and 100 mg/L), were evaluated for toxicity using a Microtox analyzer (Fig. 4). The consequences showed that yellow sweet corn grains (116.5 EC50) were significantly less toxic than the white variety (126.1 EC50).

Different concentrations of KFeO_2_-NPs significantly impact on the content of toxicity in the grains of sweet corn. The highest toxicity content was recorded in conventionally grown sweet corn (135.5 EC50) as compared to the various concentrations of KFeO_2_-NPs.

Similarly, it was observed that the interaction between varieties of sweet corn and concentrations of KFeO_2_-NPs significantly affects the toxicity concentration in sweet corn grains. The best treatment in terms of toxicity concentration was observed with yellow sweet corn grains resulting from treating the plant with KFeO_2_-NPs at a concentration of 100 mg/L (102 EC50) compared with other treatments.

All samples demonstrated non-toxic behavior, with effective concentration (EC₅₀) values ≥ 100, indicating that they are safe for human consumption and environmentally friendly. These findings suggest that the application of KFeO_2_-NPs in the tested concentrations poses no toxicological risk.

### Genomic DNA analysis using ISSR markers

**Fig. 5:**
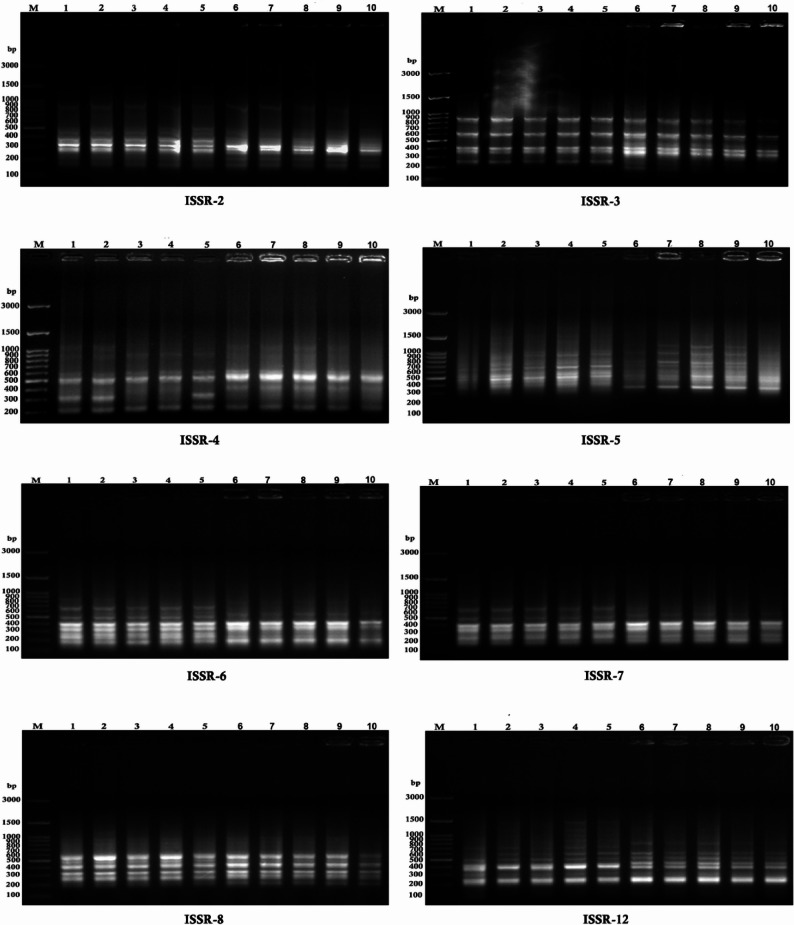
The effect of KFeO_2_-NPs concentrations on DNA profile for the sweet corn plant by using ISSR markers. Note: M**;** DNA ladder (100 bp), white corn (1: control, 2: 25 mg/L, 3: 50 mg/L, 4: 75 mg/L, 5: 100 mg/L) and yellow corn (6: control, 7: 25 mg/L, 8: 50 mg/L, 9: 75 mg/L, 10: 100 mg/L)

Eight ISSR primers were used to assess DNA fingerprint variation and GTS% in white and yellow corn treated with various concentrations (25, 50, 75, and 100 mg/L) of KFeO_2_-NPs compared to the control group (Fig. 5; Tables 7 and 8). The original image of ISSR markers is illustrated in Fig.S1.

**Table 7:** The effect of different concentrations of KFeO_2_-NPs on the genomic DNA of white corn by ISSR marker. (+ : present, - : absent)

Ser. No.	ISSR primers		Total number of bands	Allele size range (bp)	Mono-morphic bands	Poly-morphic bands	(+ ve) Unique bands	(-ve) Unique bands	Polymor-phism percentage	Molecular Size (bp)	Rf	Control	25 mg/L	50 mg/L	75 mg/L	100 mg/L
1	ISSR-2		9	834 − 168	8	1	0	0	11.11%	385	0.725	-	-	-	+	+
2	ISSR-3		6	880 − 255	6	0	0	0	0%	-	-	-	-	-	-	-
3	ISSR-4		7	1055 − 225	4	3	0	1	42.86%	1055	0.572	+	+	-	-	-
										395	0.815	+	+	+	+	-
										320	0.857	+	+	-	-	+
4	ISSR-5		11	1625 − 425	5	6	0	6	54.55%	1625	0.503	-	+	+	+	+
										1390	0.534	-	+	+	+	+
										1080	0.590	-	+	+	+	+
										975	0.612	-	+	+	+	+
										850	0.641	-	+	+	+	+
										780	0.660	-	+	+	+	+
5	ISSR-6		10	845 − 190	7	3	0	1	30%	845	0.663	+	+	-	-	-
										750	0.688	+	+	-	-	-
										560	0.746	-	+	+	+	+
6	ISSR-7		7	825 − 180	6	1	0	0	14.28%	825	0.663	+	+	-	-	-
7	ISSR-8		9	1115 − 220	8	1	0	1	11.11%	1115	0.586	-	+	+	+	+
8	ISSR-12		10	1430 − 250	6	4	0	0	40%	1430	0.530	-	-	-	+	+
										1150	0.573	-	-	-	+	+
										990	0.605	-	-	-	+	+
										880	0.632	-	-	-	+	+
Total		69		-	50	19	-	9	-							
Average		8.625		-	6.25	2.375	-	-	27.54%							

**Table 8:** The effect of different concentrations of KFeO_2_-NPs on the genomic DNA of yellow corn by ISSR marker. (+ : present, - : absent)

Ser. No.	ISSR primers	Total number of bands	Allele size range (bp)	Mono-morphic bands	Poly-morphic bands	(+ ve) Unique bands	(-ve) Unique bands	Polymor-phism percentage	Molecular Size (bp)	Rf	Control	25 mg/L	50 mg/L	75 mg/L	100 mg/L
1	ISSR-2	10	827 − 166	10	0	0	0	0%	-	-	-	-	-	-	-
2	ISSR-3	9	1040 − 190	6	3	0	1	33.33%	1040	0.573	+	+	+	+	-
									235	0.868	+	+	-	-	-
									190	0.897	+	+	-	-	-
3	SSR-4	6	1085 − 240	6	0	0	0	0%	-	-	-	-	-	-	-
4	ISSR-5	8	1190 − 385	8	0	0	0	0%	-	-	-	-	-	-	-
5	ISSR-6	7	785 − 200	5	2	0	2	28.57%	785	0.678	+	+	+	+	-
									635	0.721	+	+	+	+	-
6	ISSR-7	6	835 − 180	5	1	0	0	16.66%	835	0.716	+	+	+	-	-
7	ISSR-8	11	975 − 220	7	4	0	4	36.36%	975	0.614	+	+	+	+	-
									880	0.636	+	+	+	+	-
									770	0.663	+	+	+	+	-
									670	0.691	+	+	+	+	-
8	ISSR-12	7	815 − 260	6	1	0	1	14.28%	815	0.648	+	+	+	+	-
Total		64	-	53	11	-	8	-							
Average		8	-	6.625	1.375	-	-	17.19%							

In white corn, a total of 69 bands were detected across the primers, with each primer yielding between 6 and 11 bands. They have 50 monomorphic bands and 19 polymorphic bands, resulting in a polymorphism percentage of 27.54%. Primers ISSR-3 and ISSR-5 produced 6 bands (880–255 bp) and 11 bands (1625–425 bp), respectively. Primers ISSR-2 and ISSR-8 generated 9 bands each, ranging from 834 to 168 bp and 1115–220 bp, respectively. Primers ISSR-4 and ISSR-7 yielded 7 bands each, with size ranges of 1055–225 bp and 825–180 bp, respectively. ISSR-6 and ISSR-12 produced 10 bands each, ranging from 845 to 190 bp and 1430–250 bp. Several -ve unique bands were identified. Notably, ISSR-4 showed a unique band at 395 bp (RF = 0.815) that was absent only in the sample treated with 100 mg/L KFeO_2_-NPs. ISSR-6 and ISSR-8 each showed one -ve unique band absent in the control at 560 bp (Rf = 0.746) and 1115 bp (Rf = 0.586), respectively. ISSR-5 revealed six –ve unique bands absent in the control but present in all KFeO_2_-NPs -treated samples, with molecular sizes of 1625 bp (Rf = 0.503), 1390 bp (Rf = 0.534), 1080 bp (Rf = 0.590), 975 bp (Rf = 0.612), 850 bp (Rf = 0.641), and 780 bp (Rf = 0.660).

In yellow corn, a total of 64 bands were observed, with each primer generating 6 to 11 bands. They have 53 monomorphic and 11 polymorphic, leading to a polymorphism percentage of 17.19%. Specifically, primers ISSR-5, ISSR-3, ISSR-2, and ISSR-8 produced 8, 9, 10, and 11 bands, respectively. ISSR-4 and ISSR-7 yielded 6 bands each, ranging from 1085 to 240 bp and 835–180 bp, respectively. ISSR-6 and ISSR-12 produced 7 bands each, with ranges of 785–200 bp and 815–260 bp. Several -ve unique bands were also observed in yellow corn. One unique band was found in each ISSR-3 and ISSR-12, while ISSR-6 showed two -ve unique bands. Notably, ISSR-8 exhibited four (-ve) unique bands, all of which were absent in the sample treated with 100 mg/L KFeO_2_-NPs.

GTS% is a qualitative indication of mutagenesis effect; the results in Tables 9 and 10 demonstrate that increasing K-NP concentrations resulted in a reduction in GTS% in both sweet corn cultivars. In white maize, the lowest GTS% was 75.36% at concentrations of 75 and 100 mg/L KFeO_2_-NPs, but in yellow corn, the lowest GTS% was 82.81% at a concentration of 100 mg/L.

**Table 9:** Number of newly appeared (+) and disappeared (-) bands as related to control and GTS% in white corn under the effect of KFeO_2_-NPs using eight ISSR primers

Ser. No.	Primer name		Treatments				
			25 mg/L	50 mg/L	75 mg/L	100 mg/L	Control
1	ISSR-2	+	-	-	1 (385 bp)	1 (385 bp)	9
		-	-	-	-	-	
2	ISSR-3	+	-	-	-	-	6
		-	-	-	-	-	
3	ISSR-4	+	-	-	-	-	7
		-	-	2 (1055, 320 bp)	2 (1055, 320 bp)	2 (1055, 395 bp)	
4	ISSR-5	+	6 (1625, 1390,1080, 975, 850, 780 bp)	6 (1625, 1390,1080, 975, 850, 780 bp)	6 (1625, 1390,1080, 975, 850, 780 bp)	6 (1625, 1390,1080, 975, 850, 780 bp)	11
		-	-	-	-	-	
5	ISSR-6	+	1 (560 bp)	1 (560 bp)	1 (560 bp)	1 (560 bp)	10
		-	-	2 (845, 750 bp)	2 (845, 750 bp)	2 (845, 750 bp)	
6	ISSR-7	+	-	-	-	-	7
		-	-	1 (825 bp)	1 (825 bp)	1 (825 bp)	
7	ISSR-8	+	1 (1115 bp)	1 (1115 bp)	1 (1115 bp)	1 (1115 bp)	9
		-	-	-	-	-	
8	ISSR-12	+	-	-	4 (1430, 1150, 990, 880 bp)	4 (1430, 1150, 990, 880 bp)	10
		-	-	-	-	-	
The average number of differences in DNA profiles (a)			8	13	17	17	-
The number of bands picked from DNA control profiles (n)			-	-	-	-	69
GTS = 100 - (100a/n)			88.4	81.16	75.36	75.36	100

**Table 10:** Number of new appeared (+) and disappeared (-) bands as related to control and GTS% in yellow corn under the effect of KFeO_2_-NPs using eight ISSR primers

Ser. No.	Primer name		Treatments				
			25 mg/L	50 mg/L	75 mg/L	100 mg/L	Control
1	ISSR-2	+	-	-	-	-	10
		-	-	-	-	-	
2	ISSR-3	+	-	-	-	-	9
		-	-	2 (235, 190 bp)	2 (235, 190 bp)	3 (1040, 235, 190 bp)	
3	ISSR-4	+	-	-	-	-	6
		-	-	-	-	-	
4	ISSR-5	+	-	-	-	-	8
		-	-	-	-	-	
5	ISSR-6	+	-	-	-	-	7
		-	-	-	-	2 (785, 635 bp)	
6	ISSR-7	+	-	-	-	-	6
		-	-	-	1 (835 bp)	1 (835 bp)	
7	ISSR-8	+	-	-	-	-	11
		-	-	-	-	4 (975, 880, 770,670 bp)	
8	ISSR-12	+	-	-	-	-	7
		-	-	-	-	1 (815 bp)	
The average number of differences in DNA profiles (a)			0	2	3	11	-
The number of bands picked from DNA control profiles (n)			-	-	-	-	64
GTS = 100 - (100a/n)			100	96.87	95.31	82.81	100

## Discussion

In agriculture, nanotechnology has emerged as a powerful strategy for increasing agricultural productivity through enhanced nutrient-use efficiency and targeted delivery of essential elements. It significantly boosts plant growth and yield by improving the uptake of water and nutrients In this regard, this study shows that KFeO_2_-NPs function as an efficient foliar nano-fertilizer by significantly improving the growth, yield, and biochemical characteristics of sweet corn. Foliar feeding is especially beneficial because, as other crops have shown, it avoids soil-related nutrient losses and responds quickly to plant nutritional needs [41, 42], also, it is one effective method for applying nanofertilizers [43, 44].

### Cultivar-dependent response to KFeO_2_-NPs

The results showed a clear cultivar-dependent response where the optimal KFeO_2_-NPs concentration varied between cultivars. The yellow corn showed the best response at 50 mg/L, and the white corn at 100 mg/L. The results suggest that to improve efficiency and prevent possible stress, nanofertilizers applications should be customized for each cultivar. These genotype-specific reactions highlight the need to comprehend the molecular causes of these variations and enable precision nutrition control. This cultivar-specific reaction raises the possibility that genetic background may have an impact on nanoparticle absorption, translocation, and metabolic utilization. This finding is consistent with previous studies that have shown plant genotype can significantly impact the kinetics of nutrient uptake and interactions between nanoparticles and plants. Differences in morphological and physiological traits, such as root structure, leaf characteristics, and metabolic activity, likely affect how each cultivar internalizes and processes nanoparticles [45–47].

### Physiological and biochemical improvements

The increase in Chl a, Chl b, and Cart was strongly associated with improved morphological, growth, and yield of sweet corn, which is consistent with potassium’s function in activating photosynthetic enzymes and facilitating assimilate transport [48, 49]. These results confirm that KFeO_2_-NPs not only improve physiological efficiency but also enhance the nutritional quality of sweet corn grains. The increase in Chl pigments with increasing KFeO₂-NPs dose can be explained by potassium’s vital role in several physiological processes that enhance photosynthesis. Potassium activates enzymes involved in Chl formation, stabilizes chloroplast structure, and regulates stomatal function, improving CO₂ uptake and photosynthetic efficiency. Because of the high surface area and bioavailability of the fertilizer’s nanoparticles than conventional forms, KFeO₂-NPs enhance K uptake and promote higher Chl a, Chl b, and total Chl levels. Potassium also boosts the activity of nitrate reductase, an enzyme critical for nitrogen assimilation, which indirectly supports Chl synthesis and photosynthetic capacity. Overall, the rise in Chl content reflects improved physiological performance and photosynthetic capacity, leading to improved plant biomass and growth [50–52].

Potassium also supports root development and is vital for various growth and reproductive processes. It contributes significantly to essential physiological functions such as cell division, expansion, carbohydrate metabolism, nitrate reduction, and pollen tube formation. Moreover, potassium boosts plant nutritional value, improves crop quality, and enhances productivity [53]. Another contributing factor is potassium’s role in facilitating the transport of photosynthates from source to sink, which enhances yield [49, 54].

The data show that the application of KFeO_2_-NPs decreases the content of P in both cultivars compared to the control, while the N decreased in the white cultivar and increased in the yellow cultivar. The difference in the content of the N in both cultivars was related to cultivar-dependent response to KFeO2-NPs. In general, the nutrient interaction and competitive absorption are the primary causes of the decrease in P and N levels with greater KFeO₂-NPs dosages. P and N absorption at root or foliar locations may be inhibited by elevated KFeO₂-NPs concentrations, which would lower the uptake efficiency. Additionally, too much potassium may change the pH of the soil, the ionic balance, or the metabolic processes of plants, reducing the availability or internal concentration of essential nutrients. High K levels can further restrict N and P assimilation because plants frequently favor potassium uptake [55, 56]. The necessity of optimizing KFeO₂-NPs dosages to maintain balanced nutrient uptake and avoid macronutrient shortages is highlighted by these well-known antagonistic interactions.

The decrease in oil percentage in sweet corn grain with increasing KFeO₂-NPs levels can be attributed to the nutrient balance influence on lipid biosynthesis pathways. Excess K tends to promote starch and sugar accumulation rather than lipid synthesis, redirecting carbon and energy away from fatty acid pathways. High K can also disrupt the uptake of key nutrients like N and P, which are essential for lipid metabolism, further limiting oil accumulation. Thus, the decline in oil content reflects a metabolic trade-off caused by nutrient imbalance, emphasizing the need to optimize potassium doses for balanced nutrient availability and desired grain composition [55, 57].

The increases in sugars, proteins, phenolics, and flavonoids in sweet corn grains with higher KFeO₂-NP levels are mainly due to the potassium plays a crucial role in controlling metabolism, activating enzymes, and bolstering stress responses. By promoting enzymes like sucrose phosphate synthase and invertase, potassium improves the production and transport of sugars, while increased protein concentration results from better nitrogen assimilation and amino acid synthesis. In addition to its capacity to increase antioxidant activity and lower oxidative stress, potassium-driven activation of important enzymes in the phenylpropanoid pathway leads to an increase in phenolics and flavonoids [58, 59]. As nanofertilizers, KFeO₂-NPs enhance these biochemical effects by offering better foliar absorption and bioavailability than traditional K sources. Other crops treated with potassium nanoparticles have shown comparable increases in these metabolites, which have been connected to enhanced secondary metabolism, photosynthesis, and nutrient delivery. As long as concentrations are within ideal ranges to prevent nutrient imbalance, these metabolic improvements demonstrate the potential of KFeO₂-NPs to improve grain nutritional quality [60–62].

### Safety assessment and toxicity

The Microtox assay verified that grains collected from plants treated with all concentrations of KFeO₂-NP were shown to be non-toxic (EC_50_ ≥ 100), indicating their safety for food and the environment. In light of worries about nanoparticle persistence and possible bioaccumulation, ensuring non-toxicity is crucial for regulatory approval and public acceptability of nano-enabled agricultural products [63]. These results address important issues regarding trophic transmission, bioaccumulation, and nanoparticle persistence in agroecosystems. KFeO₂-NPs at agronomic levels (≤ 100 mg/L foliar) showed no discernible phytotoxicity, grain contamination, or ecological risk, in contrast to some metallic NPs that exhibit dose-dependent toxicity in Microtox and other bioassays. This is probably because of their controlled release, foliar application, minimizing soil accumulation, and plant metabolic processing [64, 65]. Similarly, Jiang et al. [63]. reported that EC₅₀ values based on Chl a and phosphate content indicated that silver nanoparticles (Ag-NPs) were less toxic than silver nitrate (AgNO₃), supporting the relative safety of nanoparticle formulations.

### Genomic stability and ISSR marker insights

Beyond traditional agronomic and biochemical evaluations, this study used ISSR markers to add molecular evidence in a new way. ISSR was considered an effective technique for determining genotoxicity under abiotic stress and nanoparticles effect. Numerous studies have demonstrated that nanoparticles, including Mn, Mo, Ca, Zn, K, P, and Ag-NPs, affect genetic stability and gene expression in various plant species such as common bean, sweet corn, faba bean, lettuce, and onion [66, 67]. Also, Coşkun [68, 69] mentioned that genomic stability increases due to the effect of grafting under salinity and drought conditions in Cucumber.

The use of ISSR marker analysis offers valuable insights into the genetic modifications and molecular processes underlying the observed improvements in plant traits after the application of KFeO_2_-NPs. When plants are treated with KFeO_2_-NPs, changes in gene expression may occur as part of the plant’s adaptive response. These alterations, which can include shifts in gene regulation or minor genomic rearrangements, can be detected as polymorphisms using ISSR markers. For example, a study on onion plants demonstrated that foliar application of varying KFeO_2_-NPs concentrations led to detectable molecular changes identified by ISSR analysis [51]. This suggests that KFeO_2_-NPs influence not only physiological and biochemical functions but also trigger genetic responses, potentially activating stress-related genes or modifying gene expression patterns to enhance growth and stress tolerance.

More broadly, nanoparticles like KFeO_2_-NPs can exert both beneficial and potentially harmful effects on plants. They may stimulate the production of secondary metabolites essential for plant defense and improve metabolic activity. However, excessive concentrations or unsuitable nanoparticle types may lead to the production of high amounts of ROS, that responsible for DNA damage through disrupting DNA repair mechanisms, known as necrosis or apoptosis (kill plant cells) [70].

In this study, the negative unique bands detected using eight ISSR primers in both white and yellow corn cultivars indicate that KFeO_2_-NPs may alter genomic DNA by inducing changes or substitutions at primer binding sites. These changes can result in the loss or emergence of specific bands. These findings are consistent with those of Atienzar and Jha [71], who explained that the emergence of new bands can result from modifications at primer binding sites, leading to new annealing patterns and possibly promoting homologous recombination. The differences observed between the two sweet corn cultivars in terms of growth, yield, chemical composition, and genetic characteristics can be attributed to their underlying genetic variation. The variability reported across all aspects of the study may also be linked to the genotoxic effects of KFeO_2_-NPs on genomic DNA, as indicated by the appearance of new bands in PCR analyses using different primers [72].

The GTS% is a qualitative indicator of the mutagenesis impact. It is directly proportional to the degree of DNA modification and the ability of DNA repair and replication. Genomic instability involves structural differences such as higher base pair mutation frequency [71, 73]. Our findings indicate that as the proportion of KFeO_2_-NPs used rose, the GTS% declined in both sweet corn cultivars examined.

Overall, KFeO_2_-NPs are a promising strategy for sustainable sweet corn production, according to the combined agronomic, biochemical, and molecular evidence. Before widespread implementation, however, long-term research on genetic stability and safety throughout several generations is necessary, as shown by the genomic changes found.

## Conclusions

This study shows that foliar application of KFeO_2_-NPs significantly improves the growth, yield, photosynthetic pigments, and biochemical quality of sweet corn, with cultivar-dependent optimal doses (50 mg/L for yellow and 100 mg/L for white). According to the integration of ISSR marker analysis, higher KFeO_2_-NPs concentrations reduce GTS%, suggesting possible genotoxic effects in addition to the agronomic benefits. Importantly, toxicity tests supported that grains from treated plants were safe for consumption, supporting their suitability for agricultural use. The novelty of this work lies in that it provides a thorough assessment of KFeO_2_-NPs in sweet corn by integrating agronomic, biochemical, and molecular evaluations. These results demonstrate KFeO_2_-NPs’ potential as sustainable nano-fertilizers while highlighting the significance of dose adjustment in striking a balance between genomic safety and productivity benefits. However, further studies are recommended to validate genetic stability across generations and ensure environmentally responsible application.

## Supplementary Information

Supplementary Material 1

## Data Availability

No datasets were generated or analysed during the current study.
