# Supplementary material for: Morpho-physiological and biochemical responses and genomic stability of sweet corn (Zea Mays L. saccharata) to potassium ferrite nano-fertilizer
Source: BMC Plant Biol. 2026 Feb 12;26:380. doi: 10.1186/s12870-026-08131-7 (PMC12930636; doi:10.1186/s12870-026-08131-7)
Supplement: Supplementary file 1 — Supplementary Material 1 [file 12870_2026_8131_MOESM1_ESM.docx]

 ISSR-2



 ISSR-3


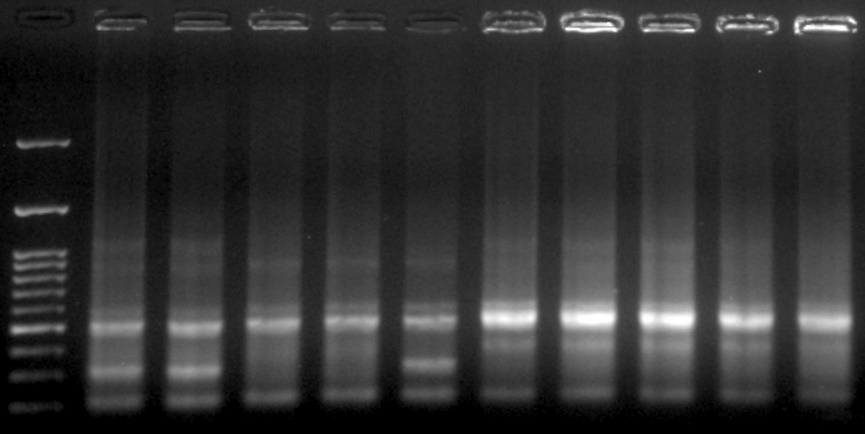
 ISSR-4


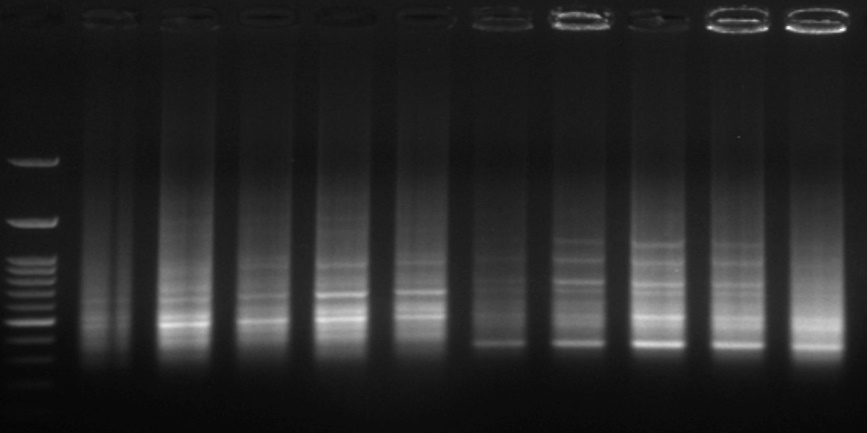
 ISSR-5


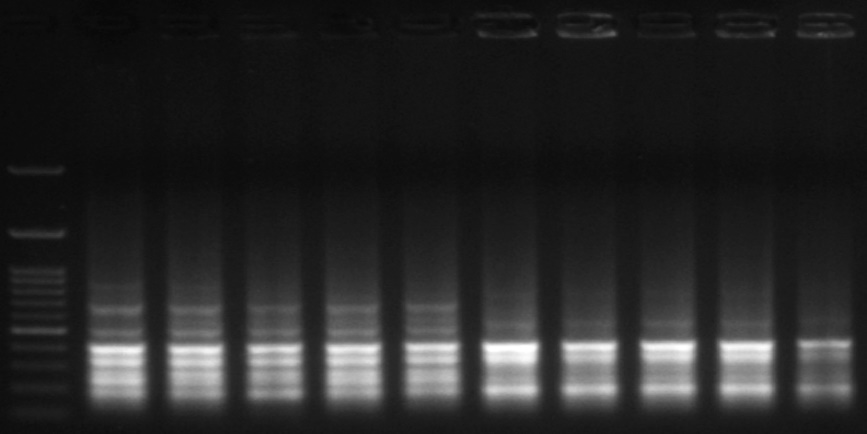
 ISSR-6



 ISSR-7


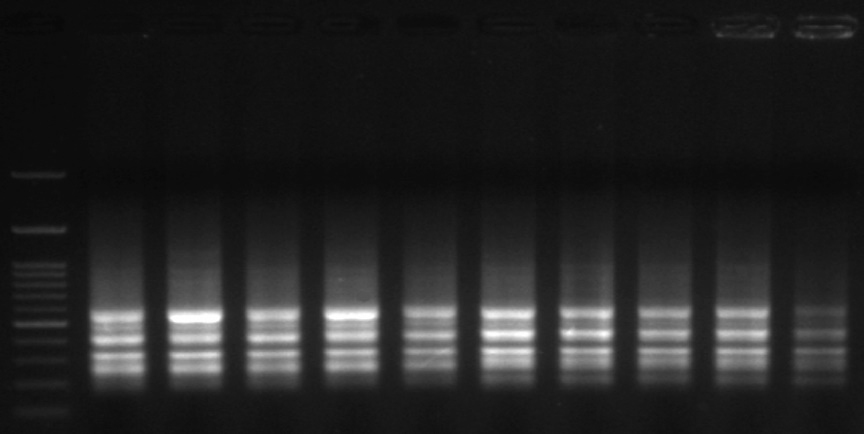
 ISSR-8


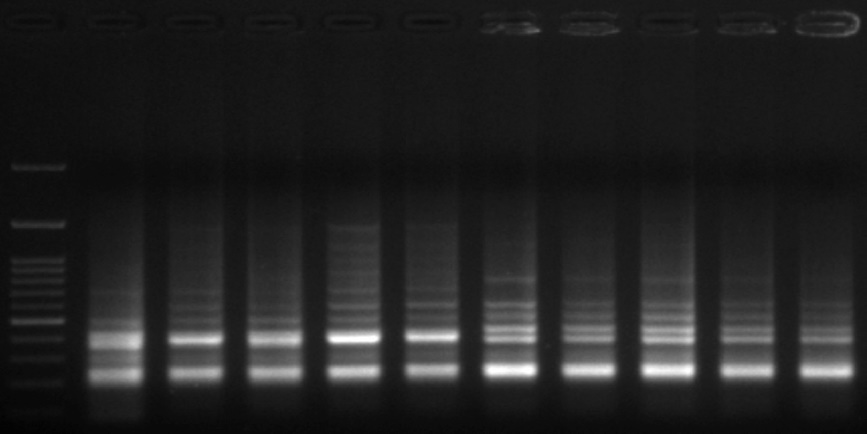
 ISSR-12

**Fig. S1:** The original figure of ISSR markers that illustrated the effect of K-NPs concentrations on DNA profile for sweet corn plant.
